# Chronic pain self-management support with pain science education and exercise (COMMENCE): study protocol for a randomized controlled trial

**DOI:** 10.1186/s13063-015-0994-5

**Published:** 2015-10-14

**Authors:** Jordan Miller, Joy C. MacDermid, David M. Walton, Julie Richardson

**Affiliations:** School of Rehabilitation Science, McMaster University, 1400 Main St. W, Hamilton, ON L8S 1C7 Ontario Canada; School of Physical Therapy, Western University, London, Ontario Canada

## Abstract

**Background:**

Previous research suggests that self-management programs for people with chronic pain improve knowledge and self-efficacy but result in negligible effects on function. This study will investigate the effectiveness self-management support with pain science education and exercise on improving function for people with chronic pain in comparison to a wait-list control. A secondary objective is to determine which variables help to predict response to the intervention.

**Methods/Design:**

This study will be an unblinded, randomized controlled trial with 110 participants comparing a 6-week program that includes self-management support, pain science education and exercise to a wait-list control. The primary outcome will be function measured by the Short Musculoskeletal Function Assessment - Dysfunction Index. Secondary outcomes will include pain intensity measured by a numeric pain rating scale, pain interference measured by the eight-item PROMIS pain interference item-bank, how much patients are bothered by functional problems measured by the Short Musculoskeletal Function Assessment - Bother Index, catastrophic thinking measured by the Pain Catastrophizing Scale, fear of movement/re-injury measured by the 11-item Tampa Scale of Kinesiophobia, sense of perceived injustice measured by the Injustice Experience Questionnaire, self-efficacy measured by the Pain Self-Efficacy Questionnaire, pain sensitivity measured by pressure pain threshold and cold sensitivity testing, fatigue measured by a numeric fatigue rating scale, pain neurophysiology knowledge measured by the Neurophysiology of Pain Questionnaire, healthcare utilization measured by number of visits to a healthcare provider, and work status. Assessments will be completed at baseline, 7 and 18 weeks. After the 18-week assessment, the groups will crossover; however, we anticipate carry-over effects with the treatment. Therefore, data from after the crossover will be used to estimate within-group changes and to determine predictors of response that are not for direct between-group comparisons. Mixed effects modelling will be used to determine between-group differences for all primary and secondary outcomes. A series of multiple regression models will be used to determine predictors of treatment response.

**Discussion:**

This study has the potential to inform future self-management programming through evaluation of a self-management program that aims to improve function as the primary outcome.

**Trial registration:**

ClinicalTrials.gov NCT02422459, registered on 13 April 2015.

## Background

Approximately 19 to 29 % of Canadians, Americans and Europeans experience chronic pain [1–3], and pain-related disability is the largest contributor to years lived with disability [4]. Pain related-disability has an important impact on the quality of life, workplace productivity and the healthcare system [5–7]. It is important, therefore, to investigate strategies to improve quality of life and reduce pain-related disability for people living with chronic pain.

Self-management refers to an individual’s “ability to manage the symptoms, treatment, physical and psychosocial consequences and life style changes inherent in living with a chronic condition. Efficacious self-management encompasses ability to monitor one’s condition and to effect the cognitive, behavioural and emotional responses necessary to maintain a satisfactory quality of life.” [8] Self-management support aims to increase participants’ skills and confidence in managing their health through the provision of education and supportive interventions.

Self-management programs commonly evaluated in the literature have included education on a number of self-management strategies and often an opportunity to practice these skills: problem solving, communication with health care providers, use of healthcare resources including medication, general stretching, strengthening, and aerobic exercise, goal setting, diaries, self-monitoring, relaxation, symptom management strategies and cognitive strategies to help cope with pain [9, 10]. Evidence on the effectiveness of self-management support for people experiencing chronic pain is limited. Most research investigating the impact of self-management support on pain and disability include people with either arthritis or low back pain [9]. The evidence suggests self-management support improves knowledge and self-efficacy, but does not produce clinically important effects on pain or function [9–11]. It is not clear whether these results generalize to more diverse populations of people with chronic pain.

Two treatment approaches for people with chronic pain that have not been included in traditional self-management programs and demonstrate improvements in function are pain neurophysiology education and individualized, goal-oriented exercises. Pain neurophysiology education has been defined as an educational intervention and is useful for “describing the neurobiology and neurophysiology of pain, and pain processing by the nervous system” [12]. Pain neurophysiology education is effective for individuals with chronic low back pain [12–14], whiplash-associated disorder [15] and chronic fatigue syndrome [16]. The influence of pain neurophysiology education on pain and function in other chronic pain conditions has yet to be investigated with a rigorous trial. Similarly, while most self-management programs encourage participation in exercise and physical activity; most have not included individualized exercise programs despite evidence of reduced disability for both musculoskeletal and neuropathic pain conditions with these approaches [17–21]. The intervention evaluated in this study will be self-management support that incorporates individualized exercises and pain neurophysiology education with a primary aim of improving function.

It is also not clear from previous research which persons are most likely to respond to chronic pain self-management support. Previous research has suggested a number of factors that may contribute to chronic pain and reduced functional rehabilitation outcomes. For example, high initial pain levels [22–24], female sex [23], lower expectations of recovery [25], low pressure pain thresholds [26], cold hyperalgesia [27–29], catastrophic thinking [30–32], sense of perceived injustice [33] and fear of movement or re-injury [34–36] have all been associated with chronic pain, disability, or poor rehabilitation outcomes. Some of these variables have been suggested as prognostic indicators with more consistency than others. This study will investigate whether some of these prognostic indicators help predict response to chronic pain self-management support with pain education and individualized exercise.

### Objectives

#### Primary objective

1. This study will test the hypothesis that participants with chronic pain experience greater improvement in function over 18 weeks with 6 weeks of Chronic Pain Self-management Support with Pain Science Education and Exercise (COMMENCE) in comparison to a wait-list control.

#### Secondary objectives

1. This study will test the hypotheses that people with chronic pain experience greater pain intensity, pain interference, self-efficacy, catastrophic thinking, fear of movement/re-injury, pain neurophysiology knowledge, bother by difficulty with functional activities, fatigue, depressive symptoms, healthcare utilization and work status with COMMENCE in comparison to a wait-list control after 18 weeks.
2. This study will compare the change in outcomes demonstrated by the wait-list group during their treatment period (18 to 36 weeks) to the change demonstrated during the wait-list period (0 to 18 weeks).
3. This study will estimate whether the impact of the intervention is maintained over an intermediate follow-up term (18 to 36 weeks) in the treatment group.
4. This study will determine whether the estimate of the magnitude of the effect is influenced by an 18-week delay.
5. This study will identify demographic, psychological or psychophysical variables that are predictive of treatment response.

## Methods/Design

### Study design

**Fig. 1:**
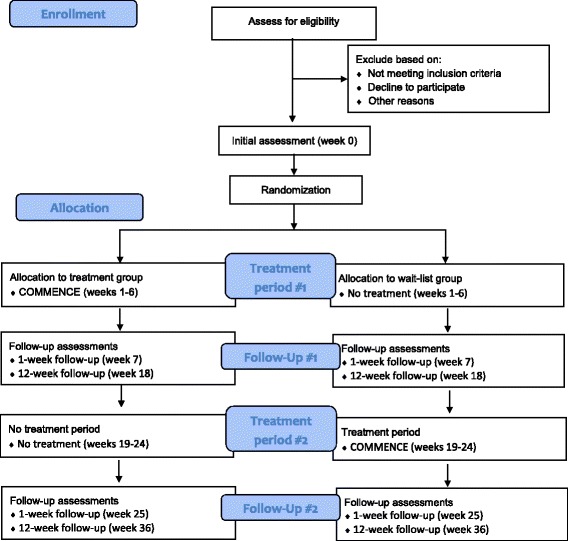
Study flow diagram

This study is a randomized trial with two parallel groups. Participants will be allocated in a 1:1 ratio to treatment and wait-list groups. After the 18-week assessment (after the 6-week treatment period and the 12-week follow-up), the group initially receiving COMMENCE will receive no treatment, and the group initially on the wait-list will receive COMMENCE for 6 weeks. Both will be assessed again at 25 and 36 weeks from the baseline (1 and 12 weeks after the wait-list group finishes treatment) (See Fig. 1 for study flow). During the treatment, wait-list or follow-up periods, participants can continue with usual care with their family physician.

Between-group comparisons (objectives 1 and 2) will be limited to the 0- to 18-week period prior to the wait-list group receiving treatment. However, an 18- to 36-week period was added for ethical reasons (that is, the wait-list group will receive the treatment) and to allow four additional analyses to address objectives 3 to 6.

### Blinding

Due to the nature of the treatment and comparison, participants and the treating physiotherapist will not be blinded to group allocation. The assessor who is completing the two objective measures will be blind to the treatment allocation at all assessment time points. The investigator performing the analysis will be blinded to the treatment allocation.

### Participants and setting

For the purposes of this study, 110 participants with chronic pain will be recruited at Woodstock and Area Community Health Centre (WACHC) in Woodstock, Ontario, Canada. All participants will be referred to the program by a healthcare provider at WACHC. WACHC has an interdisciplinary team of healthcare providers that work collaboratively to provide primary care, health promotion and community development programs to priority populations in Oxford County, Ontario, Canada. Since participants will be referred from WACHC, they will meet at least one of the criteria for WACHC’s priority populations: addictions concerns, mental health challenges, low incomes, lack of health insurance, or isolated seniors. Therefore, this sample will include people often excluded from research and treatment by barriers to accessing healthcare.

Included participants will all have been experiencing noncancer related chronic pain. Chronic pain will be defined as occurring in anyone who has been experiencing pain for greater than 12 weeks. The pain can fluctuate in intensity, but the patient must report experiencing pain on a daily basis over the 3-month period. The presentation can be that of musculoskeletal pain or neuropathic pain and can be associated with a traumatic (for example, injury or surgery) or nontraumatic etiology (for example, degenerative changes or pain of unknown etiology). Exclusion criteria will include cancer related pain, medical “red flags” suggestive of a non-neuromusculoskeletal etiology of symptoms, casted fracture or surgery within the last 26 weeks and evidence of upper motor neuron lesion. “Red flags” could include unremitting night pain, palpable tumor, sudden weight loss or weight gain, bowel or bladder incontinence, saddle anaesthesia, bilateral or multisegmental loss of sensory or motor function, fever/chills, diplopia, dysphagia, dysarthria, drop attacks, or nystagmus.

### Sample size

The sample size necessary for a randomized controlled trial with three repeated measures at 0, 7 and 18 weeks was calculated using online sample size software (GLIMMPSE 2.0) using methods detailed by Muller et al. [37–39]. The calculation was performed using a significance level of 0.05, a power of 0.8, a minimum detectable mean difference between groups of 10 points on the Short Musculoskeletal Function Assessment Dysfunction Index (SMFA-DI) [40] at both 7 weeks and 18 weeks, and a standard deviation at each time-point of 23 points on the SMFA-DI based on baseline data from a series of 20 people with chronic pain referred to physiotherapy at WACHC. The calculations were made assuming a correlation between baseline and 7 weeks of 0.84, a correlation between baseline and 12 weeks of 0.82, and a correlation between 7 weeks and 18 weeks of 0.84, based on the same clinical population. The needed sample size calculated was 88 participants. To account for a potential 20 % drop-out rate, the investigators will recruit 110 participants (n = 55 in each group).

### Allocation

The allocation sequence will be generated by a study investigator (JMD) who is not involved in the enrollment of participants or assigning interventions. A computer-generated blocked random number schedule will be used to determine allocation sequence. The block size will be unknown to the other study investigators. Participants will only be assessed and randomized if agreeable to participating in the group that starts one week after the assessment. If participants are unavailable for the upcoming group, both the assessment and randomization will be deferred. The allocation sequence will be concealed through the use of sequentially numbered, opaque envelopes, which will be opened by the physiotherapist (JM) and communicated to the patient after the initial assessment is completed.

### Enrollment

Patients will be screened and enrolled by the single treating physiotherapist (JM) after receiving a referral from a healthcare provider on the WACHC interdisciplinary team. The healthcare providers at WACHC will be instructed to refer anyone with noncancer related pain for at least 3 months. The physiotherapist will then screen to determine whether the participant meets the inclusion or exclusion criteria for participation in the trial.

### Intervention/treatment

COMMENCE consists of two visits with a physiotherapist per week over 6 weeks. One of the two visits is in a group setting, where the emphasis is on pain science education and self-management strategies using cognitive behavioral principles to support behavior change. The second visit is an individualized, one-to-one session focused on providing support to implement self-management strategies and develop an individualized, goal-oriented exercise program. Both the individual sessions and the group sessions will be carried out by a single physiotherapist (JM) for all participants.

#### Group pain neurophysiology and self-management education

The group sessions will include two to six people. The reason for a maximum of six people is pragmatic due to the maximum number of individual appointments the physiotherapist can accommodate in his schedule at the community health centre. The treatment group will proceed with as few as two people in case of low recruitment or high drop-out rate. The group sessions will be interactive 1.5 hour sessions once per week over 6 weeks. The participant will be educated on science of pain [12, 41] including the function of the nervous system, other systems involved in the pain experience, changes in these systems when pain persists, neuroplasticity, and self-management strategies to apply the information learned with the goal of increasing physical activity and participation in life role activities while controlling symptoms. The self-management strategies included in this study are informed by evidence as well as self-efficacy theory and social cognitive theory [42–45]. Self-management strategies will include progressive goal setting [46–48], activity scheduling [49, 50], thought monitoring [51], relaxation [52], sleep education [53], reflection [51], self-monitoring [10], graded activity [53–55] and exercise [19, 20]. The self-management education has been designed with the priority populations in mind. Lower average income is one of the priority populations, and this is associated with lower levels of education and literacy [56]. The material will be targeted toward those with less than a high school education. Participants will be given a workbook that guides them through the self-management strategies including goal setting, activity scheduling, using an activity and exercise log, thought monitoring and graded activity planning. It will be reviewed between the physiotherapist and participant at each individual session to facilitate communication, encourage discussion regarding an individual implementation plan for each of the self-management strategies discussed in the group, and to provide an opportunity to review any material covered in the group session.

#### Individualized self-management and exercise

The individualized sessions will be pragmatic 30 to 45 minute sessions once per week. The content will be tailored to the individual and delivered by the same physiotherapist (JM) who delivers the group sessions. The individualized sessions will include developing an implementation plan for the self-management strategies discussed in the group session. Participants will also collaborate with the physiotherapist on a graded activity plan to work towards functional goals and individual exercises to improve functional abilities to facilitate achieving functional goals. There will be three types of exercises encouraged. The first (i) is frequent pain-free movement, four to six times per day, six to ten repetitions at a time. Participants collaborate with the physiotherapist to find simple movements that can be performed easily throughout their daily routines. The purpose of these exercises is to reduce sensitivity to movement and build confidence with movement that does not increase pain. The second (ii) are exercises that simulate functional tasks needed to perform goals, one to two times per day at an intensity that allows the individual to perform eight to 15 repetitions at a time. The purpose of these exercises is to increase functional abilities needed to resume participation in life-role activities and participation goals set by the participant. Education regarding progression will be provided frequently throughout the program. The third (iii) is regular aerobic exercise. Participants will choose any aerobic activity they would like to participate in, set a baseline volume and intensity for that activity and create a plan with the physiotherapist for progression over time. The volume and intensity will be determined using recommendations that participants do not need to avoid pain at the time of exercise, but should choose an intensity that does not result in pain 1 to 2 hours after exercise. All three types of exercise will be delivered with messaging consistent with self-management education that suggests exercise is an effective way to manage pain and to prepare for increases in participation in physical activity and life-role activities.

#### Co-intervention

Participants will be free to continue with other treatments. Other treatments will be recorded through self-report at each assessment time-point and analyzed for between-group differences.

### Wait-list control

The wait-list control will be waiting to participate in COMMENCE after the 18-week assessment, and participants will be free to continue with “usual care.” This includes continued use of prescribed medications and recommendations from other healthcare providers. The wait-list comparison was chosen rather than a more robust comparator due to previous evidence, suggesting no difference in function when comparing other self-management programs to no-treatment or usual-care control groups.

### Withdrawing participants from this study

Participants may withdraw from the treatment at any time. Participants who choose to withdraw will be documented, and data will be analyzed as a member of the group to which they were randomly assigned.

### Ethical clearance

All participants will provide voluntary written informed consent after a discussion about what study participation entails and the potential benefits and risks. Informed consent will be obtained by the treating physiotherapist (JM) prior to the initial assessment after receiving a referral from a health care provider for each potential participant. Ethics approval has been obtained from Hamilton Integrated Research Ethics Board (HIREB #13-472).

### Outcomes

#### Self-report measures

All self-report measures have been demonstrated to be reliable and valid in a population of people with persistent pain. The ranges of each scale, as well as the minimal change considered clinically meaningful for this study, are described in Table 1.

**Table 1:** Outcome measures and potential predictors of response

Construct	Outcome measure	Scale range	Minimal important difference
Function	Short Musculoskeletal Function Assessment - Dysfunction Index (SMFA-DI)	34–170	10 points^a^
How much participants are bothered by difficulty with functional activities	Short Musculoskeletal Function Assessment - Bother Index (SMFA-BI)	12–60	5.5 points^a^
Pain intensity	Numeric Pain Rating Scale (NPRS)	0–10	2 points [86]
Fatigue	Numeric Fatigue Rating Scale (NFRS)	0–10	1.4 points [87]
Pain interference	PROMIS Pain Interference Item Bank - 8 items	8–40	5 points^a^
Depressive symptoms	Patient Health Questionnaire - 9 (PHQ-9)	0–27	5 points [88]
Catastrophic thinking	Pain Catastrophizing Scale (PCS)	0–52	38 % of scale [89]
Fear of symptom exacerbation	11-item Tampa Scale of Kinesiophobia (TSK-11)	11–44	5.6 points [90]
Pain neurophysiology knowledge	Neurophysiology of pain test (NPT)	0–13	1.1 points^a^
Self efficacy	Pain Self Efficacy Questionnaire (PSEQ)	0–60	11 points [91]
Work status	Working versus not-working		
	Working full hours versus modified hours		
	Working full duties versus previous duties		
Healthcare utilization	# of health care visits during 12 weeks prior to treatment versus health care visits during 12-week follow-up period		
Post-traumatic stress symptoms	Post-traumatic Stress Disorder Checklist	17–85	
Sense of perceived injustice	Injustice Experience Questionnaire (IEQ)	0–48	
Medication use	Number of medications		
	Medication by class		
Comorbidities	Disease count		
Cold sensitivity	A novel test of cold sensitivity		
Pressure sensitivity	Pressure Pain Threshold (PPT)		

Legend: This table depicts each construct being measured as either an outcome or potential predictor of response, the measure used to evaluate that construct, the range of the scale (if applicable), and the minimal important difference for scales that will be measured as outcomes

^a^In the absence of an established MCID or MDC, change greater than half a standard deviation will be considered clinically meaningful [92]. In these instances, clinical data from Woodstock and Area Community Health Centre was used to establish the standard deviation

The primary outcome will be function as measured by the Short-Musculoskeletal Function Assessment - Dysfunction Index (SMFA-DI) [40].

Secondary outcomes will include the following:

1. Short Musculoskeletal Function Assessment - Bother Index (SMFA-BI) [40]
2. Numeric Pain Rating Scale (NPRS) [57]
3. Numeric Fatigue Rating Scale (NFRS) [58]
4. PROMIS Pain Interference Item Bank - 8 items [59]
5. Patient Health Questionnaire (PHQ-9) [60–62]
6. Pain Catastrophizing Scale (PCS) [30, 63, 64]
7. Tampa Scale of Kinesiophobia - 11 (TSK-11) [65]
8. Pain Self Efficacy Questionnaire (PSEQ) [66–68]
9. Neurophysiology of Pain Questionnaire (NPQ) [69]
10. number of health care visits
11. work status

Potential predictors of response will include baseline measures for each of the outcome measures listed above as well as:

1. Post-traumatic Stress Disorder Checklist (PTSD-C) [70, 71]
2. Injustice Experience Questionnaire (IEQ) [33]
3. number of medications [72]
4. disease count [72]

#### Demographic information

The following information will be collected at the initial assessment and analyzed as potential covariates and predictors of response: age, sex, work status prior to symptom onset, length of time since symptom onset in months, diagnosis provided by a medical professional as reported by the patient, medication use, previous treatment received and expectations for recovery. Expectations for recovery will be assessed with two questions: i) Do you think your pain will improve? and ii) Do you think your functional abilities will improve?

#### Psychophysical measures

Two psychophysical measures will be performed in order to estimate sensitivity of the nervous system both locally (at the point identified as “most tender”) and at two standardized locations (the area of skin over the muscle belly of the upper fibers of the trapezius and tibialis anterior).

#### Pressure pain threshold

Pressure pain threshold will be measured using a handheld digital algometer (The Wagner FDX-25; Wagner Instruments, Greenwich, CT), which has been previously demonstrated to be reliable [26, 73]. The algometers will be calibrated using a known-weight technique prior to commencing the study. The algometer will be pressed perpendicularly into the skin at a rate of approximately 50 kPa/s (5 N/s). The tester will be trained to ensure ability to apply pressure consistently at this rate. Three measurements of the pressure pain threshold will be recorded for each site and on each side of the body. The pressure pain threshold will be determined using the following standardized instructions, used in a previous study investigating pressure pain threshold [74]: “I’m going to begin applying pressure to your skin. I want you to tell me the moment the sensation changes from comfortable pressure to slightly unpleasant pain.” For consistencym the more tender side will be tested first followed by the less tender side at the “most tender” location. At the standardized locations, the right side will be tested first, followed by the left.

#### Cold hyperalgesia testing

Cold hyperalgesia will be tested using a novel test. This device consists of a Peltier Cooler used to cool 2 pairs of cylinders. The two pairs of cylinders are made of acrylic and copper. When the temperature of the cooler is 0 degrees, the cylinders of different materials will feel similar to different temperatures on the skin. The acrylic cylinder will feel like 18 degrees, and the copper will feel like 0 degrees. Each of the two cylinders will be placed in contact with the skin at the three locations in the same randomized order as was used for pressure pain threshold. The order of the two materials will also be randomized with each of the two materials being placed on the skin three times on each side. The participant will be asked to rate how cold the cylinder is on a 21 point scale (0 is unable to detect the temperature, 10 is cold but not painful, 11 is cold and slightly uncomfortable, and 20 is unbearable pain).

#### Adverse events or harms

Participants will be asked by the physiotherapist at each visit about adverse events that the patient associates with treatment. Any adverse events requiring medical care beyond the scope of the treating physiotherapist will be referred immediately.

#### Treatment adherence

Treatment adherence will be assessed through a combination of attendance (categorized as < 25 %, 25 to 49 %, 50 to 74 %, or ≥ 75 % of visits) and adherence to self-management strategies. Adherence to recommended self-management strategies will be described as either completed or not completed by the clinician when reviewing the participant workbook at the individual treatment session.

#### Timeline for assessments

Assessments will take place at baseline, 7 weeks (1-week follow-up), 18 weeks (12-week follow-up), 25 weeks (1-week follow-up after wait-list group treatment period) and 36 weeks (12-week follow-up after wait-list group treatment period). See Fig. 1 for the flow diagram. Demographic information will be collected at the baseline assessment. Self-reported outcome measures will be collected at all assessment time-points. The two objective tests will be collected at baseline, 18 weeks and 36 weeks.

### Participant retention

Participant retention will be encouraged by clearly asking only those willing to commit to all assessment and treatment time points to enroll in the study. In addition, free parking is provided for all treatment and assessment visits, treatment is provided free-of-charge, and a small gift card ($20) is provided at each assessment time point to thank participants and encourage patient follow-up.

### Data collection and management

Hard copies of self-report data will be collected directly for the outcome measures previously listed and referenced. The measures will be completed at WACHC, with the treating physiotherapist and a research assistant present. Demographic data will be collected on pre-piloted study forms. Data will be transferred directly to a database by a trained research assistant or study investigator. Data quality will be assured through checking 10 % of the data entered. The data will be collected and stored using only participant codes with no patient identifiers. Hard copies of forms will be stored in a locked cabinet within a locked office at WACHC. The electronic database will be password protected and stored on a password protected computer. Only study investigators or staff will have access to the data.

### Data monitoring and auditing

This trial will not have a data monitoring committee and will not include auditing of the study conduct outside of the study investigators. This decision was made because of an estimated low risk to participants. Participants with chronic pain will be medically stable and are not expected to be at a high risk of mortality. In addition, self-management programs, exercise, and pain education have all been studied with no serious adverse events reported, so no harm to patients is expected with this intervention. There are no stop rules or preliminary analyses planned.

### Analysis

Statistical analysis will be conducted using Stata software, version 13 (StataCorp, College Station, TX, USA). Baseline characteristics for both treatment and wait-list groups will be presented as means and standard deviations for normal data, and medians and interquartile range for non-normal data, and number of patients and percentages for categorical data. Between-group comparisons will be made for baseline data using a Student’s *t*-test for continuous data and Chi squared or Fisher’s exact test for categorical variables to determine the results of the randomized allocation.

To address objectives #1 and 2, between-group differences in change in primary (SMFA-DI) and secondary outcomes will be analyzed using linear mixed-effects modelling with repeated measures at 0, 7, and 18 weeks. A *P* value of less than 0.05 will be considered indicative of statistical significance for all comparisons. The minimum changes required for the change to be considered clinically meaningful for each scale are listed in Table 1. An advantage of using linear mixed-effects modelling is the ability to utilize all available data points without multiple imputation when there are multiple missing data points [75]. Between-group comparisons will be limited to data collected before the 18-week assessment time point due to anticipated carry over effects due to the long-term changes in function associated with exercise approaches for people with chronic pain [19, 20]. Analysis will use intention-to-treat principles.

To address objective #3, a within-group analysis in the wait-list group will be performed comparing the change in function during the treatment period (weeks 18 to 36) to the change in function during the wait-list period (weeks 0 to 18) using a mixed effects model. If there is no change during the wait-list period, this analysis allows for a secondary estimate of treatment effect similar to a diamond response design [76]. Goldsmith et al. suggest that if the magnitude of the effect of the intervention is similar to that estimated through the comparison between groups during the 0 to 7 week period, then they may be pooled for a more precise estimate of treatment effect [76].

To address objective #4 and estimate whether treatment effects are maintained beyond the 18-week assessment, the functional score (SMFA-DI) at the end of treatment (7 weeks) will be compared with the functional score at the 25- and 36-week assessments using a linear mixed effects model.

To address objective #5, the influence of the 18-week delay on the treatment effect will be determined by comparing the magnitude of treatment effect from the waist list group (from objective #3) with the estimate of the magnitude of treatment effect from the treatment group (from objective #1).

In order to address objective #6, each of the patient demographics, outcome measure scores, and objective measures will be tested for univariate relationship with SMFA-DI change score at 18 weeks (difference between SMFA-DI at 18 weeks and SMFA-DI at baseline) using a Pearson r for continuous variables and Chi squared tests for categorical variables. Variables with a significance of <0.10 will be included in the multivariate analysis so that no potential predictive variables will be overlooked. Potential predictor variables will be entered into a series of multiple regression models to determine which combination of baseline variables best predict changes in SMFA-DI.

#### Sensitivity analyses

There will be two planned sensitivity analyses. The first sensitivity analysis will compare participants who attend at least 75 % (9/12) of treatment visits to the wait-list control group to gain an estimate of efficacy versus effectiveness. If there are any cases removed from analysis due to a high influence on the mixed effects model (cooks distance > 4/n) [77], a sensitivity analysis will also be performed to compare the results of the mixed effects model with and without highly influential points included.

#### Protocol modifications

Any changes to protocol will be communicated with all study investigators and the Hamilton Research Ethics Board in writing. If there are any changes, the trial registry (clinicaltrials.gov) will be updated electronically. If the risks to participants change, all trial participants will be contacted directly by phone to communicate the change.

## Discussion

A number of limitations in this protocol may contribute to a risk of bias. First, due to the nature of the intervention and comparison, the participants and the healthcare provider cannot be blinded. The primary outcome is a self-report measure completed by the participants (not blinded) and secondary outcomes are either self-report measures (not blinded) or psychophysical tests conducted by research assistants (blinded). The principle investigator (JM) is also the treating physiotherapist and will be present at the assessments. Additionally, the nature of the comparison could influence the risk of bias. Patients in the wait-list group will understand that they are not receiving the intervention under investigation, and this could bias their self-report assessments at 7 and 18 weeks.

Having a single therapist and center influences the generalizability of the results. While easily generalizable to the physiotherapist and setting in which the study was carried out, the ability to generalize the results to other settings and other settings is limited due to the potential of a therapist effect. A limitation of the current study design is that results may be attributed to either the intervention or the therapist effect without the ability to distinguish between the two potential mechanisms.

Another important factor when considering the generalizability of results is the population being studied. The priority populations at WACHC include people with addictions concerns, mental health challenges, low incomes, lack of health insurance and isolated seniors. This group experiences a number of barriers to accessing healthcare, and therefore, it is possible that attendance and adherence to the program will be low. Also, people with multiple morbidities have a lower functional status and experience greater functional decline with age [78]. This may limit the potential for functional gains in this population and may make it challenging to determine which factors are contributing to reduced function in this population. These factors make generalizability to a population experiencing similar barriers to accessing healthcare easier; however, generalizability to other populations without such barriers is more challenging.

The population of people with barriers to accessing healthcare also poses a risk of higher attrition rates. For example, people with depression, substance abuse issues, and lower education are more likely to be lost due to failure to locate [79, 80]. The investigators have put in place measures to try to minimize the attrition rate, including asking for multiple methods of contacting the participant and planning to make multiple attempts to contact the participant for follow-up appointments.

Another limitation of this study design is the short-term follow-up before the wait-list group receives treatment. Estimating whether changes in function are maintained in the longer term (up to 36 weeks) will rely on within-group analysis of the treatment group. Given the lack of comparison, these results should be interpreted with caution. Changes at 25- and 36-week follow-up could be due to lasting treatment effects; however, period effects could also contribute to any long-term changes. Despite the limitations, the investigators considered it important to estimate the longer-term changes in function to inform future research on long-term efficacy.

Similarly, the comparison of the treatment period (weeks 18 to 36) with the wait-list period (weeks 0 to 18) in the wait-list group should be interpreted with caution given the lack of comparison group. Between-group comparisons performed during weeks 0 to 18 will provide a better estimate of treatment effect; however, the secondary estimate of treatment effect can add precision to effect estimates and allows investigators to estimate the impact of an 18-week delay before starting the self-management program. It is important that within-group changes are not be compared between groups as this has the potential to be misleading [81].

Treatment of chronic pain is a challenge [82, 83]. Improving function is frequently reported as an important outcome by people living with chronic pain [84], and reducing pain-related disability is important for reducing the financial burden [5–7]. Self-management represents an important opportunity to improve pain-related disability and ultimately the impact of chronic pain [85]. Unfortunately, existing evidence suggests chronic pain self-management support does not result in substantial changes in participant function [9, 10]. This study aims to evaluate a new approach to chronic pain self-management that targets function as the primary outcome. If this approach is demonstrates effectiveness, this could inform self-management programming to include greater focus on pain neurophysiology education and physiotherapist-led, individualized, goal-oriented exercise. By determining which factors help to predict an intervention response, practitioners will have valuable information on the prognosis of participants entering the program. Future research may help to develop tailored approaches to self-management for persons less likely to respond to this approach. Ultimately, this research could help to improve self-management for people with chronic pain.

The results of this trial will be published in peer-reviewed journals and presented at international conferences in the fields of pain and rehabilitation.

## Trial status

Recruitment started in September 2013. At the time of protocol submission, this study is recruiting patients.

### Dissemination

This study will be published in a leading journal and presented at international conferences in the field of pain and rehabilitation. A lay summary of results will be sent to study participants. Additionally, study results will be disseminated to clinicians through courses, presentations and workshops to a community of practice of physiotherapists in primary health care and a network of physiotherapists interested in the treatment of pain.

## Abbreviations

ANOVA: analysis of variance
COMMENCE: Chronic Pain Self-management Support with Pain Science Education and Exercise
IEQ: Injustice Experience Questionnaire
NFRS: Numeric Fatigue Rating Scale
NPQ: Revised Neurophysiology of Pain Questionnaire
NPRS: Numeric Pain Rating Scale
PCS: Pain Catastrophizing Scale
PHQ-9: 9-item Patient Health Questionnaire
PPT: Pressure Pain Threshold
PROMIS: Patient-reported Outcome Measurements Information System
PI: pain interference
PSEQ: Pain Self-efficacy Questionnaire
PTSD-C: Post-traumatic Stress Disorder Checklist - Civilian Version
SMFA: Short Musculoskeletal Function Assessment
DI: Dysfunction Index
BI: Bother Index
TSK: Tampa Scale of Kinesiophobia
WACHC: Woodstock and Area Community Health Centre
